# Promises and Challenges of Microalgal Antioxidant Production

**DOI:** 10.3390/antiox8070199

**Published:** 2019-06-27

**Authors:** Clementina Sansone, Christophe Brunet

**Affiliations:** Stazione Zoologica Anton Dohrn, Istituto Nazionale di Biologia, Ecologia e Biotecnologie marine, Villa Comunale, 80121 Napoli, Italy

**Keywords:** microalgae, antioxidant, biodiversity

## Abstract

The exploration of natural antioxidants for nutraceuticals and pharmaceuticals industries has recently increased. This communication aims to grasp the relevance of microalgae in the panorama of natural antioxidant molecules supply to industrial applications as alternatives and/or complements to those typically used from higher plants. Microalgal richness in antioxidant compounds and scavenging ability compared to higher plants is discussed in the context of microalgal biodiversity. We mainly focus on families of powerful antioxidant compounds that have been scarcely investigated in microalgae, such as phenolic compounds, sterols, or vitamins, discussing the promise and challenges of microalgae as providers of health benefits, for instance, through their use as functional food ingredients.

## 1. Antioxidants and the Ability of Organisms to Finely Balance Oxygen between Cell Life and Death

Oxygen is essential but can be harmful for life on Earth, causing oxidative stress in cells and tissues through the development of ROS (reactive oxygen species) [1]. Lipids, nucleic acids (RNA and DNA), and proteins represent the main targets of ROS, reactive nitrogen species (RNS), and reactive sulfur species (RSS) [2].

Antioxidants, scavengers of ROS, are substances able to protect, scavenge, and repair oxidative damage, thereby protecting target structures or molecules from oxidative injuries [3]. In protecting against ROS, antioxidants help optimize human physiological functions, thus helping to maintain a healthy state and protect against diseases. Numerous compounds, such as some vitamins, carotenoids, and polyphenols (such as flavonoids), play a relevant role in preventing oxidative damages caused by free radicals by scavenging activity, and/or have a key role in the prevention of degenerative neuropathies or diabetes or in preventing cardiovascular diseases or cancers, as well as exerting anti-inflammatory, anti-viral, or anti-ageing activities [4,5,6,7,8,9]. 

The antioxidant endogenous machinery in humans, although highly efficient, is not enough by itself to counteract the development or harmful effects of ROS, thus requiring a supplement of exogenous antioxidant molecules. Indeed, recent studies report that human longevity is also related to the ingestion of food with high content of antioxidants, which help in protecting the body against ROS [10]. Exogenous antioxidants are mainly derived from photosynthetic organisms and belong to different families such as polyphenols (phenolic acids, flavonoids, anthocyanins, lignans, and stilbenes), carotenoids (xanthophylls and carotenes), sterols, or vitamins (vitamins B, D, E, and C) [11]. Some of them are only synthesized in vegetables and bio-accumulate in animals [10] and along ecosystem trophic web, such as in marine systems [12]. The sea is a rich source of antioxidants, such as vitamins B_12_, C, D, E, peptides, amino acids, chitooligosaccharide derivatives, astaxanthin and generally carotenoids, sulphated polysaccharides, sterols, phlorotannins, phenolic compounds, and flavones [13,14,15,16,17,18]. 

Investigating new natural antioxidants for nutraceuticals and pharmaceuticals industries is a relevant key-research topic [19]; microalgae are highly promising in this context [11,12,13,14,15,16,17,18,19,20]. 

## 2. The Small Size of the Bioactive Power: Promises of Microalgae as Antioxidant Providers

Microalgae are characterized by a high biodiversity (Table 1) and richness in terms of adaptive traits allowing them to colonize all kind of aquatic ecosystems. 

The metabolic diversity of microalgae, stemming from the adaptive flexibility of the microalgal world, makes them promising candidates to be exploited in biotechnological applications [33]. The advantages of microalgae compared to higher plants or fruits—the actual main source of antioxidants for human—derive from the combination of being photosynthetic, mainly unicellular, displaying high growth rate, and occupying reduced space for their large cultivation. 

In Table 2, we report data from literature comparing the antioxidant activity of microalgae vs. higher plants or fruits. 

**Figure 1:**
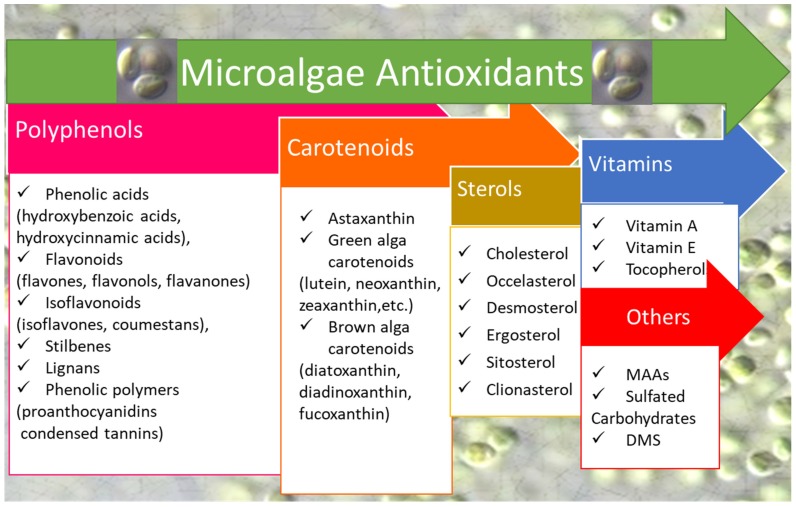
Challenging microalgal antioxidants of interest for biotechnological issues. DMS: dimethylsulphide.

The antioxidant power of microalgae is comparable, and even higher than, the antioxidative activity of higher plants or fruits (Table 2). In both cases, the variability is high (ranging from ≈4 to 260 Trolox equivalents µmol g^−1^DM). Interestingly, the antioxidant potential of some classes of microalgae such as *Chlorophyta* and *Eustigmatophyceae* (Table 2, highest values ranged from 214 to 258 Trolox equivalents µmol g^−1^DM) is comparable to the antioxidant activity displayed by *Rubus* sp. (raspberry) fruits (224 Trolox equivalents µmol g^−1^DM [34]). These results point to the reason that there is such great interest in the highly promising microalgae as antioxidant providers for nutraceuticals and human wellness, and invoke the necessity of further exploring this great potential. The relevant antioxidant activity is probably related to the high content and diversity of antioxidant molecules in microalgae, which are a source of a wide range of antioxidant molecules [42,43,44,45,46,47,48] (Figure 1), some of which are aquatic-specific, while others are shared with terrestrial plants. 

Astaxanthin, an “aquatic” carotenoid, is one of the most known for its health properties [49]. Among carotenoids, many are shared with higher plants [50], while algae (micro- and macro-) contain peculiar ones, such as fucoxanthin, which is well known for its bioactivity [51], and many others, such as diatoxanthin, diadinoxanthin, siphonein, or siphonaxanthin, with potentially interesting bioactivity [21,52]. Also, aquatic protein pigments such as phycobiliproteins are of great interest for their antioxidant and pharmaceutical activity [53]. Aquatic organisms, like microalgae, can also be providers of other sources of antioxidant molecules, such as the mycosporine-like amino acids (MAAs, [29]), which act as sunscreens against UVs and also possess antioxidant and osmoprotectant activities [54]. Moreover, the osmolyte dimethylsulphoniopropionate (DMSP) and its enzymatic cleavage product dimethylsulphide (DMS), produced in some microalgae have also been shown to display antioxidant activity [30].

Other families with powerful antioxidant activity that are well known in higher plants are also present in microalgae, although they tend to be far less studied in microalgae than in terrestrial plants (e.g., phenolic compounds, sterols and vitamins). Phenolic compounds, including several classes of flavonoids, such as isoflavones, flavanones, flavonols, and dihydrochalcones, have a protective effect on the liver, which is one of the principal targets of ROS-related diseases [55]. *Spirulina* sp., aquatic cyanobacteria, are a rich source of phenolic compounds including gallates, chlorogenates, cinnamates, pinostrobates, and p-hydroxybenzoates [56] as well as salicylic, trans-cinnamic, synapic, chlorogenic, and caffeic acids [57]. Previous studies have looked at the content and diversity of sterols in microalgae (see [16] and references therein) and have reported that microalgae can be relevant producers of sterols. Microalgal sterols have beneficial health effects in diseases such as hypocholesterolemia and neurological diseases like Parkinson illness, and also possess anticancer and anti-inflammatory activities [17]. 

Together with phenols and sterols, microalgae are also a rich source of vitamins, such as vitamin E (tocopherols), D, and C, as well as β-carotene (pro-vitamin A), pyridoxine, nicotinamide (vitamin B_3_), thiamine (vitamin B_1_), riboflavin, and biotin [58]. Sulfated polysaccharides isolated from microalgae also display relevant antioxidant properties with effective scavenging abilities on superoxide radicals, hydroxyl radicals, and hydroxyl peroxide [59]. Furthermore, microalgae are also a rich source of protein enzymes, peptides, and amino acids [60], which are necessary for the normal physiological activities of cells and tissues and have strong health-protecting effects [60]. 

## 3. *BioDivAct* (Biodiversity and Bioactivity): A Microalgal Antioxidant Challenging Project

Activation of physiological regulation pathways induced by environmental stress generates the synthesis of molecules that are able to react against ROS. These bioactive molecules are of strong interest for biotechnological applications, especially for nutraceuticals and cosmetics. Investigating natural sources of bioactive molecules and enhancing their synthetic yield are biotechnological requirements for further addressing societal needs in terms of human wellness. Marine microalgae, which represent a reservoir of known and unknown biodiversity, can majorly contribute to this goal [61]. Indeed, microalgal diversity (Table 1) offers a broad range of adaptive biological features (which may be fruitful for biotechnological applications [48]) related to their growth in the most varied marine systems, from intertidal sediments, polar or ultra-oligotrophic systems, to coastal ecosystems. Yet, a lack of information on regulative processes and their variability among biodiversity is notable, except on what is regarding the photoprotective responses such as the xanthophyll cycle and NPQ activation [62].

The challenge is to investigate the diversity of bioactive molecules and its modulation along the microalgal biodiversity scale. 

The steps to reach the “microalgal antioxidant illuminated life” are defined below:

1) Deeply investigate the content and diversity of the least known families of microalgal bioactive molecules. Phenolics, flavonoids, and vitamins (A, B, C, D, and E) have been scarcely documented in microalgae [16,17,18,19,20,23,63,64,65,66,67,68,69,70,71,72,73,74,75,76,77]. Compared to these families, microalgal sterols have been more documented thanks to the pioneering works of Volkman [16,63]. These groups of biotechnologically appealing bioactive molecules require deep investigations in microalgae. Also, other promising bioactive molecules such as the mycosporine-like amino acids [29,78] have to be further investigated.
2) Deploy a large screening of the little-known antioxidant molecules/families among the microalgal biodiversity, as recently conducted by Volkman on microalgal sterols [16]. One of the aims of this action is to generate a crossed biodiversity (*BioDivAct*) matrix providing information on the relationship between taxa/groups and the concentrations and relative contributions of the diverse families. From this, the “key molecule concept” can be proposed for the different microalgal groups (i.e., with “key” defined as molecules present in high quantity in cells, or by their high and/or peculiar bioactivity interest.).
3) Understand the role of these key molecules and their place in cells (e.g., chloroplasts, mitochondria, etc.).
4) Decipher the main biosynthetic pathways of these molecules.
5) Assess and compare the antioxidant activities of single molecules or families harvested from the microalgal diversity. These data must thus be included in the *BioDivAct* matrix. It is expected that some single molecules or subfamilies of molecules display greater activity of scavenging and/or repairing than others, as it is generally found in higher plants/fruits.
6) Investigate the regulative properties driving the synthesis of bioactive molecules in the different microalgal groups in relation to the functional groups they belong. This approach was already carried out by Dimier et al. [79] on the xanthophyll cycling pigments modulation with respect to the ecosystem properties where the microalgae come from. This can be done on cells grown under different environmental conditions, mainly by manipulating light (intensity, daily light dose, spectral composition [16,23,78]), or through others forms of manipulations [16], such as temperature, salinity, nutrient concentrations, and water movement during cultivation. Spectral light modulation, mainly varying the red:blue ratio, is of great interest for manipulating microalgal physiology and regulative properties [23].
7) Assess the antioxidative power of mixes of molecules/families harvested from mono-microalgal cultures. This feature is relevant since one way to enhance bioactivity concerns the synergism between different molecules/groups extracted together [80].
8) Optimize protocols to maximize the harvested yield of the targeted compounds and investigate procedures to maximize the extraction efficiency of bioactive mixtures from microalgae.
9) Investigate the biological and environmental conditions for developing the co-cultivation of different microalgal groups in order to provide an efficient complementary of the bioactive molecules.

The microalgal antioxidant challenge, with the specific aims described previously, will enhance the added value of microalgal harvested biomass in terms of bioactivity and thus its role in nutraceutics and/or cosmetics. Indeed, this will help to lower the cost of the production rate for obtaining a high-quality biomass. The costs of microalgal growth for cosmetics or nutraceutical applications (i.e. using them for bioactive compounds) have not yet been estimated. Attempts on comparing the costs of microalgal production vs. terrestrial plants production have been carried out [81], especially for energetics application, such as lipid production. The results of the previous study suggested that the production of algal biomass can be profitable, compared to higher plants, but requires a maximization of yields, and an optimization of harvesting and processing strategies for microalgal cultivation and for the enhancement of biomass quality (e.g., antioxidant richness for cosmetics or nutraceutical applications).

## Figures and Tables

**Table 1:** Principal marine microalgal classes (ca. 50,000 known species, estimated to be 200,000–800,000 species [21]; microalgal biomass represents ca. one-quarter of the total vegetation biomass in the world) and their potential in antioxidant biotechnology.

Classes	Species Number Estimation	Distribution	Forms	Known Interests for Bioactive Families	Applications	Expectations
Bacillariophyceae	10,000 [22]	ubiquitous	single, filament colonial	carotenoids	little	polyphenols, vitamins [23]; sterols [16]
Chlorophyceae	8000 [21]	ubiquitous	flagellate single	PUFAs, carotenoids	yes	
Cyanophyceae	2000 [24,25]	oligotrophic, coastal	filament, colonial single	phycobiliproteins proteins carotenoids	yes	vitamin B12 [26] vitamin E [27]
Dinophyceae	1500 [28]	ubiquitous	flagellate	sterols [16]	little	MAAs [29]
Prymnesiophyceae Pavlovophyceae	500 [21]	ubiquitous	single, filament flagellate	DMSP	little	DMSP [30] carotenoids sterols [16,31]
Crysophyceae	400 [21]	mostly ubiquitous	filament, colonial single	-	no	
Cryptophyceae	200 [32]	ubiquitous	Often flagellate	phycobiliproteins	little	
Prasinophyceae	100 [21]	mostly ubiquitous	flagellate	-	no	
Pelagophyceae	10 [21]	oligotrophic, coastal	single	-	no	
Bolidophyceae	15 [21]	oligotrophic	single	-	no	

PUFAs = polyunsaturated fatty acids; MAAs = mycosporine-like amino acids; DMSP = dimethylsulphoniopropionate.

**Table 2:** Antioxidant activity (Trolox equivalents, µmol (g^−1^DM)) of different higher plants and microalgal classes.

Species	Trolox Equivalents µ mol (g^−1^DM)	References
*Rubus* sp.	~224.80	[34]
*Rosmarinus sp.*	~116.00	[35,36]
*Zataria multiflora* Boiss	~108.00	[35,37]
*Perlagonium graveolens* L’Hér.	~36.00	[35,38]
*Chamaemelum nobile* L.	~7.60	[35,39]
*Achillea wilhelmsii* C. Koch	~3.00	[35,40]
*Carthamus tinctorius* L.	~1.80	[35,41]
Eustigmatophyceae	46.16–258.20	[42,43]
Chlorophyceae	5.50–214.34	[42,43,44]
Xanthophyceae	~122.52	[42,43]
Cryptophyceae	30.44–110.42	[42,43]
Pavlophyceae	24.19–94.19	[43,45]
Euglenoidea	~86.99	[42,43]
Different classes of Rhodophyta	16.61–67.95	[42,43]
Chrysophyceae	~57.35	[42,43]
Bacillariophyceae	4.55–48.90	[42,43,44,45,46]
Cyanophyceae	2.40–38.90	[42,43,44,45,46,47]
Dinophyceae	2.20–6.30	[42]
